# In Search of a Value Proposition for COVID-19 Testing in the Work Environment: A Social Marketing Analysis

**DOI:** 10.3390/ijerph191912496

**Published:** 2022-09-30

**Authors:** Claudia Parvanta, Alberto J. Caban-Martinez, Naciely Cabral, Cynthia K. Ball, Kevin G. Moore, Adrienne Eastlake, Jeffrey L. Levin, Dalia E. Nessim, Matthew S. Thiese, Paul A. Schulte

**Affiliations:** 1 College of Public Health, University of South Florida, Tampa, FL 33612, USA; 2 Department of Public Health Sciences, Leonard M. Miller School of Medicine, University of Miami, Miami, FL 33136, USA; 3 Department of Occupational and Environmental Medicine, School of Medicine, University of Texas at Tyler, Tyler, TX 75708, USA; 4 Department of Biosystems and Agricultural Engineering, Ferguson College of Agriculture and the College of Engineering, Architecture and Technology, Oklahoma State University, Stillwater, OK 74078, USA; 5 National Institute for Occupational Safety and Health, Centers for Disease Control and Prevention, Cincinnati, OH 45226, USA; 6 Department of Family & Preventive Medicine, School of Medicine, University of Utah, Salt Lake City, UT 84108, USA; 7 Advance Technologies and Laboratories International, Gaithersburg, MD 20878, USA

**Keywords:** COVID-19, COVID-19 testing, SARS-CoV-2, social marketing, qualitative data analysis, National Occupational Research Agenda (NORA) sector, worksite health promotion

## Abstract

Background: This study examined employer experience with SARS-CoV-2 (COVID-19) asymptomatic testing through a social marketing lens. Social marketing uses commercial marketing principles to achieve socially beneficial ends including improved health and safety behavior. Method: Twenty employers across 11 occupational sectors were interviewed about implementation of COVID-19 testing from January through April 2021. Recorded transcripts were coded and analyzed using marketing’s “Four P’s”: “product,” “price,” “place,” “promotion.” Results: COVID-19 tests (product) were uncomfortable, were easily confused, and didn’t solve problems articulated by employers. Testing was not widely available or didn’t line up with shifts or locations (place). The perceived price, which included direct and associated costs (e.g., laboratory fees, productivity loss, logistical challenges) was high. Most crucially, the time to receive (PCR) results negated the major benefit of less time spent in quarantine and challenged employer trust. A potential audience segmentation strategy based on perceptions of exposure risk also emerged. Conclusions: This social marketing analysis suggests ways to improve the value proposition for asymptomatic testing through changes in product, price, and placement features in line with employers’ expressed needs. Study findings can also inform creation of employee communication materials that balance perceived rewards of testing against perceived risks of exposure.

## 1. Introduction

Workers are frequently required to follow health and safety guidelines such as wearing personal protective equipment. Even when such practices are regulated, those who believe the benefits of performing the behavior outweigh the “perceived costs,” such as, restrictions of movement or breathing, tend to be more compliant [1,2,3]. Social marketing has been used successfully to tip the decisional balance toward voluntary adoption of protective behaviors in infectious or chronic disease prevention [4,5,6,7,8]. By contrast, social marketing has not been used widely in occupational safety and health (OSH) interventions [9]. Notable exceptions have been in farming and ranching safety [10,11,12], construction [13,14], and in some Total Worker Health interventions [15,16,17]. Hence, there is precedent for using social marketing in OSH, but its true potential has not been realized.

This paper is an example of how social marketing can be applied to COVID-19 testing as a primary means to return workers safely to on-site jobs. The study uses research conducted by the authors, who were either fulltime staff of, or Intergovernmental Personnel Act (IPA) assignees to, the U.S. Centers for Disease Control and Prevention (CDC)/National Institute for Occupational Safety and Health (NIOSH) at the time of the study. The Employer Testing of COVID-19 History (ETCH) study involved in-depth interviews with 20 employers representing companies in 11 National Occupational Research Agenda (NORA) sectors, all employee size categories, and multiple states. The interviews explored how diverse organizations managed their response to COVID-19, with an emphasis on testing and screening measures. Interviews were conducted from January through the end of April 2021, when the death toll attributed to the virus climbed to 500,000 in the United Sates. The transcripts from these interviews have been analyzed twice. The first analysis [18] used thematic coding based on responses to the questions asked. As a co-author on that study, this paper’s first author noted that the interview transcripts provided rich details about employer experiences with COVID-19 testing and their perceptions of products, processes, costs, and benefits. With a background in social marketing, she recommended a re-analysis of the transcripts using social marketing criteria to provide additional insights to guide both messaging around testing, as well as future OSH interventions.

### Commercial Marketing and Social Marketing

A compact definition of commercial marketing is solving a problem with a product or service for a price [19]. Successfully marketed commercial products solve problems for millions of people at what they consider a reasonable exchange of money or other things they value. *Social* marketing applies strategies and tactics learned from commercial marketing to influence the voluntary adoption of socially beneficial behaviors [20]. While financial profit is downplayed in social marketing the “price” is made up of whatever sacrifice the consumer makes to acquire the new behavior, which may be psychological.

Social marketing has more than 50 years of evidence led by practitioners such as Dick Manoff [21], Bill Novelli [22], Bill Smith [23] and Craig Lefebvre [24], as well as academic scholarship [1,25,26,27,28]. Lefebvre and Flora [24] and others [20,29] laid out key elements, or benchmarks, that distinguish a social marketing approach from other equally well-intentioned approaches, such as health education, or international social mobilization. (See Supplemental Table S1 for “Social Marketing Benchmark Criteria”).

Among the benchmarks, use of the “Marketing Mix” is most fundamental [30]. The marketing mix is referred to by commercial and social marketers alike as the “Four P’s” and includes “product”, “price”, “place”, and “promotion,” as elaborated below:

- **Product.** The first step in any form of marketing is recognizing a problem that potential consumers can articulate (e.g., need to clean teeth), or that taps into a more latent desire (e.g., be attractive to others). The second step is to then create a product that solves this problem. Third, the product needs to be distributed and promoted so that potential customers can find it if not by a brand (e.g., “Crest”) then by a category (e.g., toothpaste). The product identity is comprised of a name or brand, its inherent attributes (e.g., flavor, fluoride, whitening ability in toothpaste), as well as its potential benefits to consumers (e.g., taste, cavity protection, whiter teeth). The marketer must also be aware of the competition (other brands in category) or substitutions (other product categories) that potential consumers might choose to solve the perceived problem. In *social* marketing the “product” is often a behavior change [30]. For example, naming a designated driver has been a socially marketed behavior change to reduce driving under the influence. The “product” is the benefits perceived for performing this behavior, which in the case of the designated driver, is social approval by peers. A COVID-19 example is wearing a face covering correctly. The “product” would include the mask attributes (its material, construction, fit); the behavior of wearing it; and the perceived benefits, which have been debated (protecting self; protecting others).
- **Price**. This is anything given in exchange for the product, which in commercial marketing is almost always money, but in social marketing more often includes time, energy, a sacrifice of comfort or pleasure (as in the examples above) or going against social norms when a negative behavior is normative [31]. A COVID-19 example would be having to endure mocking from peers for wearing a face mask correctly when social norms have shifted to not wearing face coverings.
- **Place.** The location to acquire a product or service, or where a behavior will be performed. The goal is to make the offering easy to find and access [32].
- **Promotion**. This is the communication strategy used to support the full intervention [33]. In commercial marketing, promotion may include incentives such as cents off coupons or gifts with purchase.

A social marketer works with all elements in the marketing mix, and not only the promotional strategy, to develop a socially beneficial product or service that meets the needs and desires of potential consumers.

## 2. Materials and Methods

### 2.1. Parent Study

The parent study [18] consisted of a mixed-methods sequential design to identify facilitators and barriers for SARS-CoV-2 testing among U.S. businesses. In phase one of the study, semi-structured interviews were completed with 20 individual employers to explore how diverse worksites were managing their response to COVID-19, with an emphasis on testing and screening measures. Employers were located across the United States and spanned 11 industry sectors as represented in the National Occupational Research Agenda (NORA) [32]. Firms ranged in size from under 20 employees, to large, multi-facility/multi-state organizations. Insights gained during phase one were then used to refine questions for a quantitative survey sent to a large sample of U.S. companies during phase two, in progress. This social marketing analysis is a secondary analysis of the phase one qualitative transcripts using a different coding scheme, as explained below.

### 2.2. Participant Recruitment and Eligibility Criteria

The project team, supported by CDC’s National Institute for Occupational Safety and Health (NIOSH), used its existing professional networks to identify company owners, upper-level managers, human resource leaders, or health and safety officers for in-depth interviews. Eligibility criteria for participation in the study were adults ≥18 years of age who speak and write in English and work for, or own, a U.S. business principally operating in the United States. Company leaders of all races and ethnicities were encouraged to participate, but these factors together with their identities were not disclosed to the interviewers. Interviewees were not provided any compensation for participation in the study.

### 2.3. In-Depth Interviews

Twenty interviews were conducted from January through April 2021 using a virtual meeting platform that allowed for audio recording and transcription of the interview [33]. Participants were instructed to leave their name blank when they logged onto the platform and to not transmit their video image to provide privacy and confidentiality in the study. Project team members followed a semi-structured interview script [18], with most interviews running under 30 min. The generated transcripts were reviewed by the team member conducting the interview, and transcription errors were corrected before forwarding to other team members for thematic coding.

### 2.4. Initial Phase One Data Analysis

The transcribed documents were analyzed with computer-assisted qualitative data analysis software [34] and an initial coding scheme [31,35,36] based on the interview topic guide. The corpus of verbatim transcripts included 2318 “open” coded segments which, following several rounds of thematic recoding, [37,38] produced twelve emergent themes such as “Perceived risk of exposure to SARS-CoV-2,” “Guidance followed for testing,” and “Return-to-work policies.” Coded quotations were sorted into 12 thematic matrices. Respondents were identified by occupations within NORA sectors (e.g., Healthcare, Retail, Mining, Education, Manufacturing). These 12 matrices and a template for further analyses were shared with the ETCH research team to complete the parent study [18].

### 2.5. Analysis for Social Marketing Themes

Using the ‘word search’ feature of the software, the first author examined the structured data set for respondent quotations using the terms “test or testing,” “access/accessibility,” “cost(s),” “benefit(s),” “challenge(s),” “promotion,” and “communication”. This approach helped pinpoint discussions germane to social marketing criteria in each respondent transcript. The entire research team was asked to examine one or more of the prepared code matrices and create a value proposition considering their knowledge of, and experience with, one or more occupational sectors. Table 1 shows the questions given to the research team to guide the value proposition analysis.

## 3. Results

Results are presented below by the “Four P’s” along with interpretative summaries and illustrative respondent quotes. The participating employers are identified by their occupation sector labels and their ID number in the code matrices (e.g., Mining 14, Healthcare 2). The ID numbers have been retained to maintain connection to data (available upon request).

### 3.1. Product

#### 3.1.1. Product Identity

As mentioned in the introduction, a product must solve a problem that the intended user identifies for themselves. Only one respondent working in healthcare described specific problems that COVID-19 testing addressed, saying, “*We don’t have supply enough to do screening of employees, we only have enough to do testing of symptomatic people.*” In other words, the first requirement for effective marketing—definition of a problem by potential consumers—was articulated by only 1 out of 20 interviewees.

The way participating employers solved the problem of returning workers safely to work was by relying on entry-based screening for fever and self-reported symptoms. In marketing terms, they made ‘substitutions’ that competed with the product that public health authorities had designed to solve the product (second marketing step), but it was misaligned with the consumer’s view of the problem.

Illustrative quotations (shown by the sector and ID number in the data set):

*(Healthcare 6) “The return-to-work policy changed over time. Initially, it was test based and then, as we realized that you could test positive for a really long time and still not really be sick. We flexed to a time-based strategy based on the CDC guidelines.”*

*(Transportation 17) “It’s self-reporting as far as coming to work. They are asked if they are sick not to come into work and they have to self-report if they’re not feeling well.”*

*(Healthcare 2) “Definitely self-reported, and then … nursing supervisors check temperatures at a certain location.”*

The third marketing step is making sure potential consumers can correctly identify the product. When non-healthcare respondents described testing as a laboratory procedure, they often confused methods. Brand names, which tend to be easier to grasp, were not mentioned. Instead, tests were referred to as ‘antibody test,’ ‘rapid test,’ or ‘PCR (Polymerase Chain Reaction)’ test, but these names were not always correct (based on participant descriptions), nor used consistently in other responses given by the same individual.

#### 3.1.2. Product Attributes

A laboratory test has tangible attributes, which, in the case of COVID-19 could include method of collection (nasal swab, saliva, blood), sensitivity and specificity, processing time, or packaging. The process of testing brings in other attributes, such as trained staff, temperature controls on the testing location, hours of operation, etc.

Based on the word search, the top three desired attributes in COVID-19 tests were: (1) accuracy/precision, (2) rapid results (<24 h preferred), and (3) non-invasive (saliva, non-painful nasal swab). Other attributes that were mentioned include: Dichotomous (yes/no) results, easy to administer, self-administered/home based, and having a clear decision algorithm for what to do with a positive or negative test result.

Several participants expressed dissatisfaction with product performance, such as painful administration, false positives, or delays in receiving testing results.

*(Manufacturing 8) “They wanted to do the … rapid test but because of my research and what I had read online and had discussed with my own rheumatologist … there was too many false positive and too many false negatives [with the rapid test].”*

*(Coal Mining 14) “It went from timely results for tests at one point we were waiting a week to get results. To honestly, … I just didn’t trust the testing at all. And employees didn’t trust it. And an employee, just case in point, there was tested three times and couldn’t get a consistent result. That becomes a major frustration.”*

*(Retail 10) “The testing is no fun. I mean nobody really wants to go get that shoved up their nose …”*

#### 3.1.3. Product Benefits

Unlike attributes, which are features of the product or service itself, potential users see benefits that reflect their perceptions of the problem(s) and how the product helps to solve them. The perceived benefits of post-exposure/asymptomatic testing from the perspective of the study employers included the following: (1) a negative test decreases time spent in quarantine post exposure and not working; (2) testing prevents the spread of COVID-19 among coworkers, family, friends, and community members; (3) there is an associated decrease in medical costs, and (4) the benefit of “decreased indemnity and the perception of safety and service to customers.” One participant (Coal Mining 15) mentioned that testing “allowed us to track the virus in the community,” but this epidemiological perspective was expressed only once.

### 3.2. Price

In social marketing, price encompasses both monetary costs for the product as well as perceived burdens of time, energy, as well as social and psychological capital to engage in the process. Dealing first with the monetary (laboratory) costs for screening tests, participants thought $1 to $15 per test could be managed, with $1 preferred. Particularly for those companies able to support working from home, testing was expected to be done by personal healthcare providers and paid for by individual health insurance.

Weekly testing was seen as affordable both in terms of laboratory costs and time required. For diagnostic testing, the cost of a medical visit, or ‘less than $50.00’ was considered appropriate.

*(Manufacturing 16): “Costs are a factor, because we are a small company … And I mean I would get some of the new stuff that comes out the ‘home testing kits’ … That might be feasible depending on the CPPs (costs per person).”*

*(Retail 7): “For 2100 employees there’s a pretty significant cost. We spent pretty significant dollars on setting up all the protocols within the store and labor.”*

*(Healthcare 6): “We can’t do mass testing of our employees. We don’t have supply enough to do screening of employees, we only have enough to do testing of symptomatic people.”*

*(Education 12): “In the beginning, there was some apprehension with the testing process. [If employees] tested positive what would happen to confidentiality? Or would [testing] be [performed during] … work-time or personal-time?”*

*(Coal Mining 14): “I fear that [testing] would be price gouging.”*

#### Associated Costs of Testing

By far the biggest perceived associated costs were additional burden on workers who were not sick and the loss of productivity while waiting for test results. Employers were also cognizant of the extreme burden of lost wages for employees who were not eligible for the federal emergency paid sick leave benefit.

*(Manufacturing 8): “So, waiting for those test results to come back it’s difficult to find replacements … We … had guys that had worked 18–19 days straight, while the other guys were in quarantine … that’s one reason we couldn’t do random [testing] on our shift people, [like] we could do on the salary people.”*

*(Transportation 17): “We’re one of the organizations with the emergency paid sick leave was able to be implemented. So, employees that weren’t feeling well they were able to … use up to those 80 h and they didn’t have to use their accrued sick leave.”*

*(Construction 20): “The major thing with the employees has been you know, ‘Am I going to be paid?’ … most of our field staff are hourly, so once that happens to them that that is their main issue. Our office staff not as much because they are on salary and as long as they make a phone call a day you know we have to pay them their full salary.”*

The mandatory post-exposure quarantine was initially 14 days, which some local jurisdictions reduced to 10 days during the study period (https://www.cdc.gov/coronavirus/2019-ncov/science/science-briefs/scientific-brief-options-to-reduce-quarantine.html, accessed 23 February 2021). When test results were not returned quickly, some employees with negative results still had to wait out the quarantine period before returning to work. This situation created an associated, and possibly lingering, cost in destabilizing trust between employers and employees who either stayed home while “waiting for test results” or came back to work without them.

*(Construction 20): For example, I could have an employee that would [say] “hey I get 80 h paid COVID pay no matter what”, so they could take a test and then tell me, “oh I don’t have the results back, I don’t have the results back and so on,’ and then almost two weeks later to the day they amazingly get [the results] back. And to be honest, I can’t judge that because I myself was one of those that I took one and, after two weeks, I still haven’t gotten the results back. Come to find out that the company messed up. So, you know I don’t want to say that my employees are trying to deceive us.*

*(Manufacturing 8): “It was just you have to follow the rules … because some people tried to sneak in, and so they felt uncomfortable having to tattle tale on their employees that … they’re sick and they’re not telling you the truth.”*

Some respondents felt their company lacked the knowledge, training, or skills to conduct employee COVID-19 testing themselves. The burden of seeking outside expertise or forming relationships with local laboratories or pharmacies was an associated cost of testing. Finally, some employers saw COVID-19 testing as a “power balance” or sensitive issue they were not willing to broach.

*(Seafood processing 19): “We have put that personal responsibility on those who feel that they need a test can go get one … If I would try to test my workers, they would quit. So, I’m not mandating testing.”*

### 3.3. Place

The social marketing concept of “place” was relevant in several circumstances: location of the business and rules to be followed for reporting of positive cases, contact tracing, and return to work guidance.

*(Manufacturing 11): “Having our business in multiple locations [meant] … we had to follow state guidelines for each of those areas, sometimes even county guidelines, and that was a little of difficult.”*

#### 3.3.1. The Convenience of Testing

*(Retail 10): “We don’t do [testing] at our store … because I don’t know how we would do that from a medical capacity …. There’s an urgent care near us that does walk in rapid test and then also through local health care provider.”*

*(Public Safety 4): “So when we had the free testing … that was given to us by the state … in which all they had to do was drive up, and it was an oral test, and so they would not even leave the vehicle. We would hand them the test and demonstrate out in front of the windshield what they needed to do. They would all do the test themselves and then hand it back to us in a box and label it and we will move on down the road.”*

*(Manufacturing 8): “We eventually found a local clinic that did testing [for us] only and you didn’t have to have symptoms … The laboratory just happens to be right in their backyard, so it went straight to the lab.”*

*(Manufacturing 5): “The smaller size sites that we have all over the states, it was not that easy … We depended on the [named national pharmacy chain]; we depended on the [named commercial lab company]; we depended on the urgent cares. We provided coverage at all these locations, so we advised our employees, you can go to your place of choice, and the nurses in most of my locations would follow until we got the results.”*

#### 3.3.2. Access to Employees to Be Tested

*(Manufacturing 16): “A bit tough given [how] spread out we are.”*

*(Retail 7): “I’m not sure how … our employees work three shifts, they’re coming and going off all times of the day and night. Some are seasonal, one day a week, and some are five days a week, they’re full time. So … Unless somebody was actually sitting in that location, all the time, how we would catch everybody?”*

### 3.4. Promotion

#### 3.4.1. Audience Segmentation

In social marketing, audience groups are defined by how potential consumers relate to the proposed product or behavior. This allows messaging to be tailored to a specific adoption stage [39], perceived benefits, or other group norms. This is considered more effective than “one size fits all” promotion. In the case of COVID-19, the respondents described conditions and concerns that present four risk perception categories, as shown in Table 2. It should be noted that these perceptual groupings are similar, but not identical to, the OSHA hazard segments (https://www.osha.gov/coronavirus/hazards, accessed 24 September 2022).

An individual’s perception of their risk of exposure to COVID-19, or transmission to others, is a critical element in the ‘value proposition’ for post-exposure testing. Referring to Table 2, those in public facing occupations such as direct patient care (Audience Segment Group 1) or public interaction (Audience Segment Group 2) perceive themselves at greater risk of exposure and transmission due to the need to mix with the public, if not touch them directly. The value they place on knowing their own COVID-19 status when asymptomatic (the individual benefit of testing) would theoretically be higher. Those in non-public facing occupations (e.g., manufacturing, mining (Audience Segment Group 3), fishing, agriculture, (Audience Segment Group 4) believe they have less risk of workplace exposure. They might conceivably value knowledge of their COVID-19 status less than groups 1 and 2, and therefore feel they have less to gain by testing. Concomitantly, the associated costs therefore appear higher. Below are Illustrative quotations to support these groupings:

Group 1

*(Healthcare 6): “As healthcare organization and given the prevalence of COVID 19 in our community and the presence of asymptomatic spread, there are pretty significant risks here, depending on where they work and what kind of work they do.”*

*(Healthcare 2): “We find you can talk to people and just continue to have positive tests for very long periods of time. And since we are in central workforce, we didn’t feel like we have the ability to just have that many people out waiting for a negative test.”*

Group 2

*(Public Safety13): “They all utilize shared computers, telephones, desk spaces, so there was concern about the sanitization or cleaning of the workstation prior to another person coming on. And similar with patrol officers, they don’t have assigned patrol cars, they share patrol cars.”*

*(Retail 7): “Initially it was talking people off the ledge. You know, a lot of concerns, a lot of fear. I think then our employees saw that … we can work safe. Within all of our stores, there was only one department in one store that ended up taking out seven people (due to COVID exposure) … and then I found out they weren’t following protocols.”*

*(Transportation 17): “Of course, they were concerned about the public. But then the mask mandate went off, so we were doing everything we could to ensure that they knew all the safety protocols and that we were doing taking extra measures to sanitize the buses, the facilities, things of that nature.”*

Group 3

*(Construction 20): “Our field staff is just, you know, scared. They are and honestly, they’re the largest population that we had [that also was at high risk for COVID].”*

*(Manufacturing-oil 8): “… We have a control room [for] the refinery … they’re enclosed in that room for 12 h or longer. They don’t leave that seat. And, then our outside crew when they’re not in the unit they’re in a blast resistant building, we call it a BRB, and so … there’s not much space there.”*

*(Coal mining 14): “At first, there was a lot of concern in regards to contact tracing and whether … they should be notified if there was a positive result and whether or not it was the company’s responsibility to notify the employees.”*

*(Coal mining 15): “Probably one of our biggest issues was being able to get people together for training purposes, not being [able] to [have] bigger groups and things like that, so that created some issues that’s one of the things we had to change up a little bit.”*

Group 4

*(Agriculture 3): “I mean … our “outside guys” naturally social distance just because of the nature of our business. It’s not like we have 50 people packed in the office.”*

*(Seafood processing 19): “For the most part, my workers, to be honest with you, think this is a farce, politically. That’s been the conversation around our dock. Those who have gotten sick with anything have just stayed home at that point and waited until they were feeling better.”*

#### 3.4.2. Promotional Strategies

Respondents also shared some ideas for ways to improve communications about testing with employees. For example,

*(Retail 7): “I developed a little flow chart, so the managers and employees kind of knew what to do. We don’t like paper, but we did mail one to every home, because we thought it was that important, so everybody knows that the protocols are for that.”*

Others suggested using corporate incentives, providing additional paid sick leave Others suggested using corporate incentives, providing additional paid sick leave hours, tax benefits, or other incentives for testing.

The idea of mandated testing (or vaccination) was controversial. For example:

*(Coal mining 14): “I also personally have a huge concern with an employer walking in and saying this is mandatory … Immunizations or vaccinations or something like that or even testing is mandatory. We don’t require you to bring your vaccination card for anything else for employment with the company, and so I personally don’t agree with that.”*

*(Public Safety 4): “They would have to be convinced that it’s necessary, I have a lot of a type A personality personnel who try to make up their own mind about things, and when it’s not absolutely mandatory that they have to do it, you’re going to get some rejection… I think the main challenge is mental. I think convincing them that it is necessary for them to all protect each other by getting tested is key. But that campaign hasn’t worked in our department as much.”*

## 4. Discussion

The Phase I qualitative study and thematic analysis for ETCH [18] described how employers were coping in general with the challenges presented by SARS-CoV-2 with a focus on testing. In our first analysis, we used grounded theory [37] to isolate key themes to capture this moment in time and isolate problems for further study in a larger scale quantitative survey. In doing this first round of analysis we saw sufficient material to re-analyze using the social marketing constructs of product, price, place, and promotion. Without additional burden to employers, a social marketing analysis could lay the groundwork for how testing products themselves could be revamped, and how SARS-CoV-2 testing as a process could be positioned to be of more value to the US workforce.

A social marketing interpretation suggests that among employers interviewed, post exposure testing of employees, regardless of symptoms appeared to be too costly and provide too few benefits to be widely adopted. There were several factors contributing to this poor value proposition for testing, beginning with the marketing cornerstone that the product was not perceived to be a unique solution that solved a problem articulated by the intended consumers. Testing as “the solution to ensuring a worker may safely return to work” came after substitutions were established. Once competition is established, marketers must do more to promote the new product’s benefits and discount its costs. For these employers, the benefits sought were “certain, quick results” which would mean “fewer employees out, and faster return to work” and therefore, “increased productivity”. COVID-19 “testing” as introduced could not deliver these benefits due to product performance issues (e.g., improper administration, inherent pain/discomfort with specimen collection, prolonged wait time for results, and perceived inaccuracies) and perceived high costs (new technologies, limited access, new partnerships required, outright medical and lab fees, associated loss of wages for daily employees).

The unalterable attribute of COVID-19 testing was its accuracy. Once this was questioned it was seen as no better, and possibly worse, than alternative practices (symptom and temperature checks)—which were perceived to be cheap, relatively easy, and generally available (once past the initial shortage of thermometers and sense that suppliers were taking advantage of this and raising prices). The desired features of the test—easy and painless, fast, accurate, cheap, accessible, and plentiful—were not available at the time [as is the case now with home-based tests.]

A second major contributing factor to the weak value proposition was the awkward nomenclature for COVID-19 tests. They were described by product *attributes*, such as the analytical target (RNA, antigen, antibody), analytical technique (PCR, immunoassay), and product benefits, “rapid test.” What employers described needing was “clear test purpose,” “clear test name,” and “simple guidelines for what to do with results”. The more uniquely defined (i.e., the “A” test is for “a” purpose; the “B” test is for “b” purpose) the better (taking a tip from direct-to-consumer marketing).

It may be possible to develop a targeted promotional strategy (i.e., communication materials, training, incentives) based on perceived risks of occupational exposure or transmission. This would reduce multiple occupations and sectors into 4 manageable audiences for testing messages and outcomes:

- Physical touching required (e.g., patient care, salon services, EMS)
- Public Facing (e.g., teaching, retail sales, commuter transportation)
- Non-Public Facing/Close interaction (e.g., manufacturing, food processing)
- Non-Public Facing/Outdoors, distanced, Working from anywhere (Fishing, agriculture, financial management).

Messages framed for these four audience segment groups might vary in how the benefits and costs of COVID-19 testing are presented.

Our findings suggest that perceived costs are an important consideration for workplace policies for COVID-19 vaccination or testing. Employers have expressed their desire to not get too involved in what they perceive to be the personal choices of their employees. Unless they are in a high risk of transmission, high risk of exposure setting, such as healthcare, employers might be reluctant to require what they perceive to be an intrusion into their employee’s private decisional space.

Finally, at the time of the study, the FDA had not yet issued emergency use authorization for antiretroviral treatments for COVID-19. The availability of oral treatments that could be given within five days of COVID-19 symptom onset in non-hospitalized patients clearly repositioned COVID-19 testing as the unique solution to a problem of patient care. Similarly, the national distribution of home-based test kits had not yet occurred, and these were in scarce supply. Both of these developments potentially raise the value proposition for COVID-19 testing for the workforce.

The limitations for this study are like many qualitative studies in that the sample size is small, and results are not generalizable. Additional limitations include recall bias of respondents and sequential coding (versus independent coding) of the data. To minimize this limitation, the data matrices were reviewed by the entire research team and are available as Supplementary Materials upon request. Interviews were completed from January through April 2021, during the third wave of the SARS-CoV-2 virus prior to the Delta variant when the U.S. death toll climbed to 500,000. Less was known about the virus and testing options at that time than we have all learned now.

## 5. Conclusions

This study is the first to apply a social marketing analytical approach to employer experiences and perceptions of COVID-19 testing, an emergency mitigation strategy for the workforce. This analysis suggests improvements are necessary in both the tangible attributes of the testing products and their delivery as well as in the framing of benefits (or value propositions) for different audience segments. Moving from formative research to pilot trials/beta testing, to large scale implementation, is standard practice for introducing new products in commercial marketing. Following the same process has resulted in numerous interventions directed to the public [4,5,6,7,8] and in worksite health promotion [40,41,42]. A plausible next step is testing the hypothetical marketing mix for COVID-19 tests (product attributes that are sought; costs considered reasonable; preferred distribution points, and promotional strategies) with different employer-based segments. The segments could be defined by perception of risk, as suggested in this analysis, or by other workplace factors that might prove more important such as specific occupations or geography. The goal would be to find common elements that resonate with different occupational groups so that myriad messages are not necessary, but sufficiently compelling for intended audiences. A social marketing plan for COVID-19 testing, or other health or safety interventions for the workforce, can be created based on such results either for ongoing use in the COVID-19 pandemic or to prepare for the next emergency event.

## Figures and Tables

**Table 1:** Questions Asked to Develop Value Proposition.

The Test (What test?)	What attributes or features are sought in a COVID-19 test? [saliva based, instant read, 24-h turnaround in lab, pre-packaged, disposable without sharps or other special containers, etc.]. What is the price point you think the sector can pay per test application? What is the normal quantity that a typical organization in this sector would need, and how often to do adequate testing?
Testing	What are the ‘benefits’ of testing for your sector? [safety of public, safety of other workers, etc.] What “place” do you think will be the most effective/efficient for reaching your sector? What do they need to ‘exchange’ to get testing done in a satisfactory manner? [loss of production, time off, days out, cost of tests, etc.] How can barriers to testing be lowered for this sector?
Competition	What are organizations within your sector doing/what did they do/ instead of using testing to return employees safely to work?

**Table 2:** Potential Audience Segmentation Based on Perceived Risk of COVID-19 Exposure and Transmission.

Audience Segment Group	Label	Examples of Specific Occupations
1	Physical contact with public required	Patient care, EMS, salon services
2	Public interaction (face-to-face) required	Teaching, Transportation, Retail, Janitorial
3	No public interaction, close inter-personal working conditions	Food processing, warehouse, apparel manufacturing, mining
4	Outside, distanced, or working from anywhere	Fishing, farming, financial management

## Data Availability

Data will be available from NIOSH when objectives of the research are complete. Please contact aeastlake@cdc.gov.

## Supplementary Materials

The following supporting information can be downloaded at: https://www.mdpi.com/article/10.3390/ijerph191912496/s1, Table S1: Social marketing benchmark criteria. References [3,20,26,29,30,39,43,44,45] are cited in the Supplementary Materials.
