# Supplementary material for: In Search of a Value Proposition for COVID-19 Testing in the Work Environment: A Social Marketing Analysis
Source: Int J Environ Res Public Health. 2022 Sep 30;19(19):12496. doi: 10.3390/ijerph191912496 (PMC9566354; doi:10.3390/ijerph191912496)
Supplement: Supplementary file 1 [file ijerph-19-12496-s001.zip › ijerph-1879670-supplementary.pdf]

Table S1. Benchmark Criteria for Social Marketing [20,29,30]

Criteria	Definition
Healthful or socially beneficial	A ***healthful or socially beneficial*** outcome at the individual or group level.
Behavior Change	A commitment to ***behavior change*** (beyond antecedent knowledge or attitude change).
Exchange	The concept of ***exchange***. For individuals to accept a new product, service, or behavior change they must be convinced of its value, because it will have a cost. Even so-called “free” offerings may require time or energy outputs or have psychological freight (e.g., the opinion of peers or family members), and these non-monetary costs are considered in the price of adoption. The “value proposition” shows how the value of the offering outweighs the costs using factors of importance to the intended users [43].
Competition	The intended user is probably already doing something else, or might use something else, instead of the beneficial behavior or product up for adoption. This ***competition*** must be considered in the marketing strategy.
Marketing Mix	The ***marketing mix*** of product features (attributes and benefits), place (accessibility and convenience), price (above) and the promotional strategy—the so called “Four P’s”— are all used to lower barriers and raise value in the mind of the intended user [26].
Research	Strategic insight comes from direct ***research*** with the intended users, about their “needs, wants, perceptions and present behavior patterns.
One size does not fit all	Audiences are segmented by psychographics more often than demographics (age, sex, ethnicity) alone, for example, by being an early or late adopter of an innovation [44]
Use of behavioral theory	E.g., Prospect Theory [3], Transtheoretical Model [39], Elaboration Likelihood Model [45].
